# Severe acute respiratory syndrome coronavirus-2 antibody prevalence in adults with HIV

**DOI:** 10.1097/QAD.0000000000004180

**Published:** 2025-03-13

**Authors:** Mary Lucey, Fiona Burns, Sanjay Bhagani, Marc Lipman, Sara Madge, Margaret Johnson, Jennifer Hart, Colette Smith, Dimitra Peppa, Tristan J. Barber

**Affiliations:** a Department of Virology; b Department of HIV Medicine, Royal Free London NHS Foundation Trust; c Institute for Global Health; d Institute of Immunity and Transplantation, University College London, London, UK.

**Keywords:** antibody, coronavirus disease, coronavirus disease-19, HIV, severe acute respiratory syndrome coronavirus-2, vaccination, vaccine

## Abstract

**Objectives:**

To measure severe acute respiratory syndrome coronavirus-2 (SARS-CoV-2) antibody seroprevalence within a cohort of adults with HIV and correlate demographics with response rates to SARS CoV-2 vaccination.

**Design:**

Initial vaccine trials for SARS CoV-2 did not examine efficacy in people with HIV. We undertook the SCAPE-HIV study from April 2021 to November 2022 to focus on vaccine response in this population to guide future vaccine scheduling.

**Methods:**

Participants completed a retrospective questionnaire. Nucleocapsid and spike antibodies to SARS CoV-2 (anti-N and anti-S) were tested. Demographic and HIV factors (CD4^+^ cell count, viral load) were correlated with quantitative serological outcomes. Anti-S titres less than 400 U/ml were considered low level. Follow-up was performed in a subset post third vaccination.

**Results:**

Six hundred and twelve participants completed the study questionnaire, 520 were included in the final analysis. Most participants received either ChAdOx1-S recombinant vaccine or the BNT162b2 mRNA vaccine for the first two doses. Almost all participants (99.2%) in the main group had an anti-S antibody detected above the assay cutoff (>0.8 U/ml). Most participants (77.3%) had anti-S titres greater than 400 U/ml, with the median titre 1734 U/ml. Age over 60 years was significantly associated with lower (<400 U/ml) anti-S antibody titre (*P* < 0.0001).

**Conclusion:**

We demonstrate a high rate of anti-S seropositivity following SARS-CoV-2 vaccination in people with HIV. Age over 60 was the only parameter found to be associated with a lower anti-S antibody titre. Our findings suggest that COVID-19 vaccine scheduling should target older persons with HIV in line with the general population.

## Introduction

People with HIV are at increased risk of acute and chronic pulmonary disease even when optimized on antiretroviral therapy (ART) [1–3]. Data collected early in the coronavirus disease-19 (COVID-19) pandemic indicated that severe acute respiratory syndrome coronavirus-2 (SARS-CoV-2) infection in people with HIV was associated with increased mortality, compared to the general population [4,5]. Although vaccination against SARS-CoV-2 reduced the severity of COVID-19 disease for most recipients, phase one mRNA vaccine trials did not report safety and efficacy data in people with HIV [6,7]. Establishing correlates of COVID-19 vaccination immunity in people with HIV is important, as previous studies in HIV have found suboptimal responses to vaccinations against influenza and hepatitis B [8,9]. Studies carried out prior to the omicron variant demonstrated robust humoral responses in people with HIV following two doses of mRNA vaccine [10–12]. Research carried out post-Omicron showed that three doses of vaccine (mRNA or adenovirus-based) were necessary to develop adequate neutralization to this variant [13]. The relationship between CD4^+^ lymphocyte count and vaccine response is less clear. Several studies demonstrated an inverse relationship between CD4^+^ cell count and anti-Spike antibody levels following vaccination [14–17]. Others reported no significant correlation between CD4^+^ cell count and serological response [18–20]. To determine the interval and duration of vaccination administration in people with HIV, it is important to identify factors associated with sub-optimal vaccine response. We designed the ‘SARS-CoV-2 Antibody PrevalencE in an adult London HIV cohort: SCAPE-HIV Study’ to investigate factors influencing vaccine response in our adult cohort of people with HIV to help inform vaccination scheduling in this group.

## Methods

SCAPE is a prospective, observational seroprevalence study undertaken between April 2021 and November 2022. Participants were recruited from the HIV outpatient department at the Royal Free Hospital, London, UK. All participants were over 18 years, with documented HIV-1 infection. Baseline data were collected through a questionnaire. Serum samples were tested for COVID-19 serology. The primary analysis looked at participants who received two SARS-CoV-2 vaccine doses at least 15 days prior to recruitment (cohort 1). A secondary analysis was undertaken in a subset of participants following a third vaccine dose (cohort 2). Recruitment was opportunistic, undertaken at routine outpatient visits.

Antibody responses to SARS CoV-2 nucleocapsid protein (anti-N) and spike protein (anti-S) were measured. Serological analysis was undertaken by the Health Services Laboratory using the Roche Elecsys Cobas e801 analyser. The Elecsys Anti-SARS-CoV-2-S Quant assay and Elecsys Anti-SARS-CoV-2N assays were used for quantitative and anti-N antibody testing, respectively. Quantitative anti-S antibody results (available for a subset of participants), were reported in units/millilitre (U/ml).

Questionnaires were stored in the local research centre. Data were transferred anonymously to electronic format. Serological results were collected from the hospital laboratory-information-management-system.

All participants had capacity to provide informed consent. Ethical approval was granted by the Royal Free Hospital London Research Ethics Committee (REC).

### Statistical analysis

Participant demographics, COVID-19 disease history and vaccine side-effects were summarized using number (percentage) and median (interquartile range). Demographics chosen reflected risk factors for severe COVID-19 infection. As all people with HIV were recommended COVID-19 vaccination, age over 60 was chosen as a cut-off to reflect older age in this population.

Antibody levels were summarized in three ways: presence/absence of antibody (binary); quantitative levels of antibody (continuous) and high vs. low/no level (≥400 U/ml vs. <400 ml; binary). Associations between quantitative antibody levels and demographic factors were assessed using chi-squared tests and Fisher's Exact test if values were low.

Antibody levels taken from cohort 2 were cross tabulated with cohort 1 to examine for statistical changes post third vaccine dose. All analyses were performed using Stata Version 15, and no formal adjustments were made for multiple comparisons.

## Results

### Baseline characteristics

Six hundred and twelve participants consented to complete the study questionnaire. Of these, 26 of 612 (4.2%) declined vaccination, 53 of 612 (8.7%) were less than15 days post vaccine, and 13 of 612 (2.1%) had no serological results available. Five hundred and twenty participants were included in the primary analysis (cohort 1). Baseline characteristics are outlined in Table 1. Most participants were white men (83.3%) with a median age of 56 years. Almost all participants (96.5%) had a suppressed HIV viral load (<40 copies/ml). The median CD4^+^ cell count was 619, and the median CD4^+^ nadir was 230 cells/mm^3^. Most received either the ChAdOx1-S recombinant vaccine (296; 56.9%) or the BNT162b2 mRNA vaccine (207; 39.8%). A small number reported vaccine side-effects (5.6%). Over two-thirds reported regularly receiving the influenza vaccine, less than one-quarter had received pneumococcal vaccination (23.9%).

**Table 1:** Baseline characteristics including self-reported coronavirus disease-2019 disease history and antibody results.

Baseline characteristics cohort 1		*n* = 520
Gender	Male	433 (83.3%)
	Female	85 (16.4%)
	Other/unknown	2 (0.4%)
	Median [years (range)]	56 (26–95)
	>60 years	156 (30.0%)
	>65 years	80 (15.4%)
Ethnicity	White	381 (73.3%)
	Black	86 (16.5%)
	Asian	23 (4.4%)
	Mixed	19 (3.7%)
	Other/unknown	11 (2.1%)
BMI (kg/m^2^) (*n* = 455)	Median (IQR, range)	25.8 (23.5, 29.6; 17.7, 51.4)
	>40	14 (3.1%)
Underlying medical conditions	Yes	264 (50.8%)
Cardiovascular comorbidity	Yes	123 (23.7%)
Respiratory comorbidity	Yes	72 (13.9%)
Renal comorbidity	Yes	32 (6.2%)
Metabolic disease^a^	Yes	66 (12.7%)
Immune/infective comorbidity^b^	Yes	26 (5.0%)
Oncological comorbidity^c^	Yes	24 (4.6%)
HIV factors		
Last HIV VL <40 copies/ml	Yes	502 (96.5%)
Last CD4^+^ cell count^d^	Median (range)	619 (134–1969)
CD4^+^ nadir^d^	Median (range)	230 (0–2993)
Current CD4^+^ : CD8^+^ ratio^d^	Median (range)	0.93 (0.03–6.15)
On ART	Yes	516 (99.2%)
ART regimen type	3-drug regimen	383 (73.7%)
	2-drug regimen	131 (25.2%)
	Other	2 (0.4%)
	Not on ART	4 (0.8%)
NRTI-containing	Yes	462 (88.9%)
NNRTI-containing	Yes	157 (30.2%)
PI/r or PI/c-containing	Yes	96 (18.5%)
INSTI-containing	Yes	310 (59.6%)
CCR5-containing	Yes	18 (3.5%)
Immunomodulatory agents^e^	Yes	14 (2.7%)
Vaccine uptake		
COVID vaccine type	BNT162b2 mRNA	207 (39.8%)
	ChAdOx1-S recombinant	296 (56.9%)
	Other	7 (1.4%)
	Unknown	10 (2.0%)
Vaccine side-effects	Yes	29 (5.6%)
Regularly receive flu vaccine	Yes	350 (67.3%)
Previous pneumococcal vaccine	Yes	124 (23.9%)
COVID-19 history		
Date of antibody testing	Median (range)	August 2021 (May 2021–September 2022)
Days since second vaccination	Median (range)	93 (0–627)
Self-reported COVID-19 infection	Yes	76 (14.6%)
	No	422 (81.2%)
	Unknown	22 (4.2%)
Hospitalized for COVID-19	Yes	6 (1.2%)
Required O_2_ therapy for COVID-19	Yes	3 (0.6%)
Qualitative antibody results cohort 1		*n* = 520
Positive anti-S antibody result	Yes	516 (99.2%)
	Titre median, U/ml (range)	1734 (0.4->2500)
Positive anti-N antibody result	Yes	135 (26.0%)
Quantitative antibody results cohort 1		*n* = 481
	Titre >400 U/ml	372 (77.3%)
	Titre >2500 U/ml	204 (42.4%)
Results cohort 2		*n* = 50
Received a third vaccine dose	Yes	50 (100%)
Vaccine side-effects	Yes	19 (38%)
COVID-19 infection (since first antibody test)	Positive PCR test	16 (32%)
Hospitalized for COVID-19	Yes	2 (4%)
Days since first antibody test	Median (IQR, range)	353 (281–401; 181–523)
Positive anti-S antibody	Yes	50 (100%)
Anti-N antibody comparison between cohorts (*n* = 50)	Positive, then positive	10 (20%)
	Negative, then negative	19 (38%)
	Negative, then positive	20 (40%)
	Positive, then negative	1 (2%)
Quantitative antibody results cohort 2		*n* = 21
Spike titre >400 U/ml	Yes	20 (95.2%)
Spike tire >2500 U/ml	Yes	18 (85.7%)

anti-N, antinucleocapsid antibody; anti-S, anti-spike antibody; ART, antiretroviral therapy; CCR5, C-C chemokine receptor type 5; CD4^+^/CD8^+^, Cluster of Differentiation 4/8; INSTI, integrase *strand transfer* inhibitors; NNRTI, nonnucleoside reverse transcriptase inhibitors; NRTI, nucleoside reverse transcriptase inhibitors; PI/r or PI/c, protease inhibitors boosted with ritonavir or cobicistat.

a The metabolic syndrome includes hypercholesterolemia, diabetes mellitus type 2, hypertriglyceridemia, obesity and hypertension.

b Current infective or immunological diagnoses other than HIV diagnosis.

c Current or past history of concomitant oncological disease.

d A CD4^+^ cell count was not available for four participants, a CD4^+^ nadir was not available for six participants and a CD4^+^ : CD8^+^ ratio was not available for 198 participants.

e Drugs that modulate the immune system such as mycophenolate mofetil, cyclosporine, tacrolimus, hydroxychloroquine, azathioprine and rituximab, not including ART.

### Antibody responses in cohort 1

The timing of first serological sampling was heterogenous with a median duration of 93 days post second vaccine dose (Table 1). Almost all participants (99.2%) in the main group had an anti-S antibody detected above the assay cutoff (>0.8 U/ml). Over one-quarter (135/520; 26%) had a positive anti-N antibody consistent with previous COVID-19 disease. Although less than 15% self-reported a history of confirmed COVID-19 disease.

Four participants failed to mount a protective antibody response. All had underlying immunocompromise (renal transplantation, primary immunodeficiency, and lymphoid malignancy).

### Antibody responses in cohort 2

Fifty participants attended for repeat serological testing following a third vaccine dose (Table 1). This visit took place a median of 353 days (range 181–523 days) after the first visit 187 days (range 31–397 days) after the third vaccination. All 50 had anti-S antibodies detected. The proportion of participants in cohort 2 who reported vaccine side-effects increased to 38%. Sixteen participants reported breakthrough infection since their last visit, 2 of 16 requiring hospitalization. Anti-N antibody status was compared between the two cohorts. Twenty participants (40%) in cohort 2 underwent anti-N antibody seroconversion indicative of breakthrough COVID-19 disease, despite receiving three vaccine doses. One participant lost their anti-N antibody, and 10 participants maintained positive anti-N antibody status. The remaining 19 participants (38%) had a negative anti-N antibody at both visits. Twenty-one participants in cohort 2 had quantitative results available. Most (95.2%) had an anti-S antibody titre greater than 400 U/ml.

### Characteristics influencing quantitative anti-spike antibody titres

Statistical analysis was carried out in 481 of 520 participants in cohort 1 with available quantitative anti-S titres (Table 2). Quantitative anti-S titres (U/ml) were compared with baseline characteristics (age, ethnicity, BMI, comorbidities and HIV-specific factors). Anti-S titres less than 400 U/ml were considered to confer lower level protection against breakthrough infection, whilst levels at least 400 U/ml were considered protective. These thresholds, albeit arbitrary, were utilized by evaluating anti-S antibody responses and risk of breakthrough infection in healthy controls and individuals with immunosuppressive conditions from prior studies and determining the upper value of the lowest decile [21–24].

**Table 2:** Characteristics influencing quantitative antibody results cohort 1 (*n* = 481).

		Spike titre ≤400 U/ml (*n* = 109)	Spike titre >400 U/ml (*n* = 372)	*P* value
Gender	Male	90 (82.6%)	313^a^ (84.6%)	0.61
Age	>60 years	48 (44.0%)	94 (25.3%)	*<0.0001*
	>65 years	24 (22.0%)	46 (12.4%)	*0.012*
Ethnicity other than white	Yes	84 (77.1%)	272 (73.1%)	0.41
BMI	Median (IQR)	25.4 (23.0–30.0)	25.8 (23.7–29.1)	0.61
	>40 kg/m^2^	4/94 (4.3%)	9/330 (2.7%)	0.45
Smoking status	Current	20 (18.4%)	68 (18.3%)	0.92^b^
	Ex	39 (35.8%)	121 (32.5%)	
	Never	50 (45.9%)	171 (46.0%)	
	Unknown	0 (0.0%)	12 (3.2%)	
Current CD4^+^ cell count	Median (IQR)	573 (401–775)	624 (457–794)	0.19
Current CD4^+^ cell count <200 cells/mm^3^	Yes	4/107 (3.7%)	10^a^ (2.7%)	0.58
Current CD4^+^ : CD8^+^ ratio	Median (IQR)	0.93 (0.65–1.21)	1.17 (0.79–1.38)	0.57
Dual antiretroviral regimen	Yes	22 (20.2%)	93 (25.0%)	0.30
Immunomodulatory agents	Yes	6 (5.5%)	8 (2.2%)	0.09^b^
Underlying medical condition	Yes	60 (55.1%)	184 (49.5%)	0.50
Taking non-HIV medications	Yes	70 (64.2%)	182 (48.9%)	0.05^b^

IQR, interquartile range.

a Gender was unknown for two participants and current CD4^+^ cell count was not available for two participants.

b Excluding unknown category in *P* value calculation.

The majority of participants (77.3%) had anti-S titres greater than 400 U/ml, with the median titre of 1734 U/ml. Age over 60 years was significantly associated with a low anti-S antibody titre less than 400 U/ml (*P* = 0.0001). Comorbidities, ethnicity, BMI greater than 40 and sex had no effect on antibody titre. Current CD4^+^ : CD8^+^ ratio and CD4^+^ count less than 200 cells/mm^3^ were not associated with anti-S titres (*P* = 0.57 and *P* = 0.58, respectively).

## Discussion

We report the seroprevalence of SARS-CoV-2 antibodies in people with HIV following COVID-19 vaccination. To our knowledge, this is the largest study of this type carried out at a single centre in the United Kingdom. Results demonstrated a strong seroconversion rate, with 99.2% of participants developing SARS-CoV-2 antibodies after two vaccine doses. We carried out multivariate analysis to identify risk factors, which predisposed to lower anti-S antibody titre, defined as less than 400 U/ml. We found age over 60 years was significantly associated with lower anti-S antibody titre (*P* < 0.0001). There was no significant relationship between quantitative antibody levels and sex, ethnicity, raised BMI or comorbidities. We did not find a correlation between CD4^+^ cell count or CD4^+^ : CD8^+^ ratio and the anti-S antibody titre. A subset of participants underwent repeat antibody sampling post third vaccine dose. All 50 had a positive anti-S antibody result. Sixteen participants reported breakthrough SARS-CoV-2 infection in between sampling, two required hospitalization. Twenty underwent anti-N antibody seroconversion between visits, indicating that four participants had asymptomatic COVID-19 disease following three vaccine doses.

Four participants did not seroconvert after the initial vaccine course. These were significantly immunocompromised in addition to their HIV diagnosis. A clinical trial comparing seroconversion rates between people postsolid organ transplantation (SOT) and people with HIV showed that people with HIV had a higher seroconversion rate than the SOT group [25].

The only variable we identified as significantly associated with lower anti-S antibody titres was age over 60 years. A study examining humoral response in people with HIV found that antibody response declined by a log_10_ (0.1) with every decade [26]. A large seroprevalence study of immunocompromised patients (but not inclusive of people with HIV) showed younger age to be significantly associated with likelihood of anti-S antibody seropositivity post vaccination [27]. The associated decline in anti-S antibody levels has been linked to an increase in comorbid conditions [28]. However, we found the association to be independent to comorbidity burden, reflecting the process of age-associated immunosenescence [29].

Multivariate analysis did not identify a significant correlation between CD4^+^ cell count and quantitative antibody titres in this group. Conversely Haidar *et al.*[17] showed that current/recent CD4^+^ cell count less than 200 cells/mm^3^ was associated with lower chance of anti-S antibody seroconversion. *et al.* demonstrated a significant relationship between CD4^+^ cell count less than 200 cells/mm^3^ and immune factors including receptor binding domain antibody strength, the level of antibody neutralization, and interferon-γ release [30]. Other studies evaluating neutralizing antibody titres found similar results in patients with CD4^+^ cell counts less than 200 and 350 cells/mm^3^, respectively [31]. These results have not been replicated consistently across the literature [26,32].

This study has several limitations. Baseline data were collected via questionnaires, which may have been prone to self-reporting bias. Most participants had a suppressed viral load and a median CD4^+^ nadir greater than 200 cells/mm^3^, which may have impacted on our ability to identify a correlation between CD4^+^ cell count and antibody response. The immune parameters collected were restricted to qualitative and quantitative anti-spike and anti-nucleocapsid antibody levels only, which provide limited insight to the complexity of immune response in people with HIV. As data collection were undertaken in an opportunistic manner at outpatient visits, intervals between vaccination and antibody testing were not consistent; therefore, we could not assess the degree to which antibody levels waned over time. Although our study lacked a control group, the sample size of over 500 participants was a significant strength. To our knowledge, there are few studies from this period, which evaluate a larger population size [19,32,33].

We noted reduced vaccine uptake rate for both influenza and pneumococcal vaccines, suggesting we may see a similar decline in SARS-CoV-2 vaccine uptake over coming years. This means more data and clearer education about the utility of vaccines for people with HIV will be needed.

In conclusion, we demonstrate a high rate of anti-spike antibody seropositivity following SARS-CoV-2 vaccination in people with HIV. This increased to 100% seroconversion following a third vaccine dose. It has been shown elsewhere that antibody levels in people with HIV waned more quickly after two doses of vaccine compared to controls [33,34]. We would, therefore, advocate for strong COVID-19 vaccination campaigns to continue on an annual basis, particularly targeting older individuals with HIV to maintain immunity in this potentially vulnerable population.

## Acknowledgements

Funding support for the SCAPE-HIV Study was provided by Roche Diagnostics and the Royal Free Charity.

Role of authors: M. L. was author of first draft and responsible for subsequent revisions; F.B. drafted study questionnaire; S.B., M.L., S.M., M.J., and J.H. provided virology expertise and contributed to study design; C.S. was the study statistician; D.P. provided immunology expertise; T.J.B. was the principal investigator and co-ordinator of the study. All authors have reviewed and approved the manuscript.

### Conflicts of interest

There are no conflicts of interest.
